# Tumor microenvironment dynamics in oral cancer: unveiling the role of inflammatory cytokines in a syngeneic mouse model

**DOI:** 10.1007/s10585-024-10306-1

**Published:** 2024-08-10

**Authors:** Ayano Tobe-Nishimoto, Yoshihiro Morita, Junya Nishimura, Yukiko Kitahira, Shun Takayama, Satoko Kishimoto, Yuka Matsumiya-Matsumoto, Kazuhide Matsunaga, Tomoaki Imai, Narikazu Uzawa

**Affiliations:** Department of Oral & Maxillofacial Oncology and Surgery, Osaka University Graduate School of Dentistry, 1-8 Yamadaoka, Suita-shi, Osaka, 565-0871 Japan

**Keywords:** Oral cancer, Tumor microenvironment, Inflammatory cytokine, Epithelial-mesenchymal transition, Syngeneic mouse oral cancer model

## Abstract

The process of cervical lymph node metastasis is dependent on the phenotype of the tumor cells and their interaction with the host microenvironment and immune system; conventional research methods that focus exclusively on tumor cells are limited in their ability to elucidate the metastatic mechanism. In cancer tissues, a specialized environment called the tumor microenvironment (TME) is established around tumor cells, and inflammation in the TME has been reported to be closely associated with the development and progression of many types of cancer and with the response to anticancer therapy. In this study, to elucidate the mechanism of metastasis establishment, including the TME, in the cervical lymph node metastasis of oral cancer, we established a mouse-derived oral squamous cell carcinoma cervical lymph node highly metastatic cell line and generated a syngeneic orthotopic transplantation mouse model. In the established highly metastatic cells, epithelial-mesenchymal transition (EMT) induction was enhanced compared to that in parental cells. In the syngeneic mouse model, lymph node metastasis was observed more frequently in tumors of highly metastatic cells than in parental cells, and Cyclooxygenase-2 (COX-2) expression and lymphatic vessels in primary tumor tissues were increased, suggesting that this model is highly useful. Moreover, in the established highly metastatic cells, EMT induction was enhanced compared to that in the parent cell line, and CCL5 and IL-6 secreted during inflammation further enhanced EMT induction in cancer cells. This suggests the possibility of a synergistic effect between EMT induction and inflammation. This model, which allows for the use of two types of cells with different metastatic and tumor growth potentials, is very useful for oral cancer research involving the interaction between cancer cells and the TME in tumor tissues and for further searching for new therapeutic agents.

**Supplementary Information:**

The online version contains supplementary material available at 10.1007/s10585-024-10306-1.

## Introduction

Cancer is the leading cause of death among people aged 30–70 years in many countries, and in recent years, it has surpassed heart disease in developed countries [1]. According to the GLOBOCAN2020 estimates, approximately 377,700 new cases and 177,700 deaths of oral cancer occur annually worldwide [2–4]. In Japan, approximately 23,600 people were newly diagnosed with oral cavity and pharyngeal cancer in 2019, and approximately 7,800 oral cancer patients died in 2020 [5].

Squamous cell carcinoma accounts for approximately 90% of all oral cancer cases, with the tongue being the most common site of occurrence [6, 7]. In oral squamous cell carcinoma (OSCC), cervical lymph node metastasis has a remarkable impact on survival; however, the mechanism of metastasis remains unclear [8]. Cervical lymph node metastasis occurs when the primary tumor enlarges and lymphangiogenesis occurs. Tumor cells invade the lymphatic lumen, survive in the lymphatic circulation, invade the cervical lymph nodes, and survive and proliferate in the new environment [9]. Each of these steps is dependent on the phenotype of the tumor cells and their interaction with the host microenvironment and immune system. Conventional research methods that focus exclusively on tumor cells are limited in their ability to elucidate the metastatic mechanism [10, 11].

In cancer tissues, a specialized environment called the tumor microenvironment (TME) is established around the tumor cells and contains a variety of non-tumor cells that play important roles in tumor development and progression. For example, stromal fibroblasts, extracellular matrix (ECM), blood vessels, lymphatic vessels, immune cells, growth factors, and cytokines secreted by TME components positively or negatively influence tumor development and progression [12–16].

Inflammation in the TME has also been reported to be closely associated with the development and progression of many types of cancer and response to anticancer therapy [17, 18]. Acute inflammation contributes to the death of cancer cells by inducing an anti-tumor immune response, while chronic inflammation has been reported to be involved in suppressing tumor immunity, providing a TME suitable for tumor formation, development, and metastasis, and inflammatory responses are sometimes induced by anticancer drug therapy [19–21]. Furthermore, advances in anticancer therapy include the development of immunotherapy, which is informed by extensive research on the intricate interplay between immune mechanisms within the TME and the tumor itself. This approach augments the anti-tumor immune response by assisting immune cells within the TME to overcome the tumor’s immune escape capacity [22]. Immune checkpoint inhibition is a form of immunotherapy that aims to induce an effective anti-tumor response in the immune mechanism [23]. Immune checkpoint inhibitors are anti-tumor agents that can inhibit immune checkpoints expressed on cytotoxic T lymphocytes (CTL) and cancer cells [24, 25]. The identification of novel immune checkpoints and their targets and the tailoring of therapy to individual patients represent two of the major goals of immunotherapy, requiring further research, the development of new combination therapies, and the development of more immunotherapeutic agents [26]. To develop novel immunotherapies, it is necessary to establish mouse models of human tumors and their microenvironment, genetically, physiologically, and anatomically, to accurately reflect human tumorigenesis and development and evaluate their efficacy [26].

Conventional in vitro experiments commonly used in cancer research, such as two-dimensional (2D) cell culture and three-dimensional (3D) organoids, are insufficient to model the complexity of the TME, which is composed of various cell types, cytokines, and growth factors [27, 28]. In our previous study, we inoculated immunocompromised nude mice with human oral cancer cell lines and established highly metastatic lymph node cell lines by in vivo selection, focusing on the association between lymph node metastasis and epithelial-mesenchymal transition (EMT) in oral cancer cells [29]. However, nude mice have a marked decrease in the number of T cells, which are important in tumor immunity, along with their dysfunction, making analysis of the TME and evaluation of immune checkpoint inhibitors targeting T cells impossible [26, 30]. In contrast, a syngeneic mouse model utilizing normal immune mice provides reproducible results in cancer immunological studies [27, 28, 31].

In this study, to elucidate the mechanism of metastasis establishment, including the TME, in cervical lymph node metastasis of oral cancer, we established a mouse-derived oral squamous cell carcinoma cervical lymph node highly metastatic cell line and generated a syngeneic orthotopic transplantation mouse model in which the TME can be observed, including the cancer immune system.

## Materials and methods

### Cells and culture

NR-S1, a mouse oral squamous cell carcinoma cell line [32], was provided by KUMAMOTO University. All cells were cultured at 37 °C under a 5% CO_2_ atmosphere in Dulbecco’s modified Eagle’s Medium Ham’s F-12 medium (D-MEM Ham’s F-12: FUJIFILM Wako Pure Chemicals Corporation, Osaka, Japan) supplemented with 10% fetal bovine serum (FBS; Equitech-Bio Inc., Kerrville, TX, USA) and 100 μg/mL kanamycin (Meiji-Seika, Tokyo, Japan). The cells were certified as free of *Mycoplasma* contamination.

### Animal model of cervical lymph node metastasis

All experiments were conducted in accordance with the ethical guidelines of the relevant institutional review board, approved by the Osaka University Graduate School of Dentistry Institutional Animal Use Committee (approval number: R-01-004-0), and followed the National Institute of Health guidelines for animal welfare.

Five- or six-week-old male C3Hf/He mice were obtained from Nippon SLC (Shizuoka, Japan). They were anesthetized using a mixture of three drugs: medetomidine (0.3 mg/kg body weight; Domitor® Nippon Zenyaku Kogyo Co., Ltd., Tokyo, Japan), midazolam (4.0 mg/kg body weight; Dormicum®, Astellas Pharma Inc., Tokyo, Japan), and butorphanol (5.0 mg/kg body weight; Vetorphale®, Meiji Seika) [26].

**Fig. 1:**
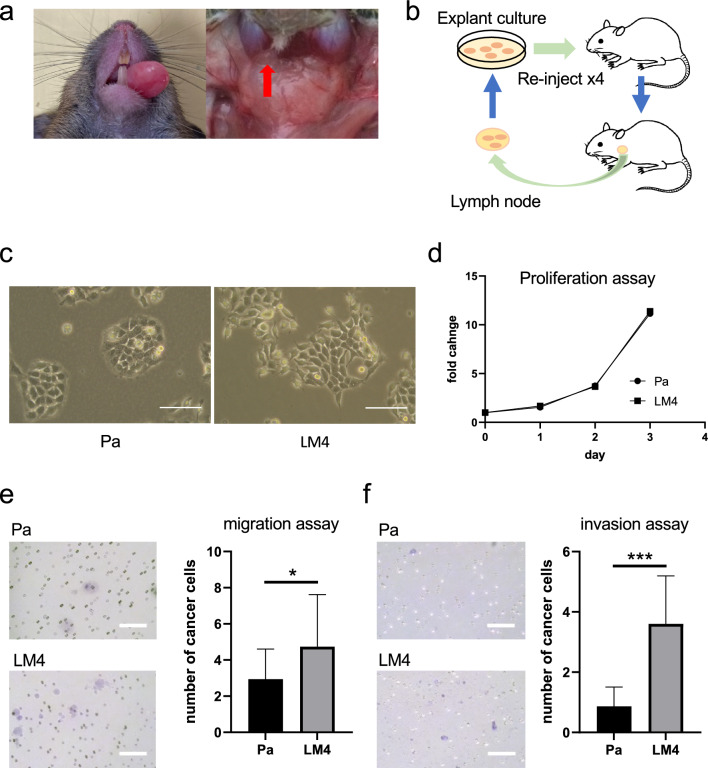
Establishment of highly metastatic cervical lymph node cell lines using in vivo selection in a syngeneic mouse model. **a** Tongue tumors and autopsied cervical lymph nodes of a normal immune mouse (C3Hf/He mouse) were inoculated with oral squamous cell carcinoma NR-S1 cells into the tongue. **b** NR-S1 cells (Pa cells) were inoculated into the tongues of C3Hf/He mice (1 × 10^6^), and cervical lymph nodes were removed after enlargement of the tongue tumors. The lymph nodes were crushed using a biomasher, seeded, and cultured in a culture dish, and the metastatic cells were passaged 2–3 times before being reinoculated into the tongues. This process was repeated four times to establish the highly metastatic cervical lymph node cell line NR-S1-LM4 (LM4 cells). **c** Representative phase contrast microscopy images of Pa and LM4 cells showing Pa cells exhibiting a paving stone-like cell morphology, whereas LM4 cells were slightly more spindle-shaped. Scale bar: 100 µm. **d** In the proliferation assay, no significant changes in cell proliferation capacity were observed between Pa and LM4 cells. Twenty-four hours incubation time was set as Day 0, and the graph is shown as a multiple of the measured O.D. (optical density) on Day 0. Values indicate means and standard deviation (n = 6). **e** In the migration assay, a significant difference in migration capacity was observed in LM4 cells compared to that in Pa cells; the number of cells was counted in fifteen fields of view, and the mean value per field of view was calculated. Scale bar: 200 µm. n=5. *p < 0.05. **f** In the invasion assay, significant differences in invasion capacity were observed in LM4 cells compared to that in Pa cells; the number of cells in fifteen fields of view were counted, and the mean value per field of view was calculated. Scale bar: 200 µm. n = 5. ***p < 0.001

Cancer cells (5 × 10^5^–5 × 10^6^ in 0.1 mL D-MEM Ham’s F-12 without FBS) were injected into the tongue apex of C3Hf/He normally immune mice. After the tongue tumor had grown, the tongue tumor and cervical lymph nodes were removed (Fig. 1a). Immediately after the procedure, euthanasia was performed.

### Establishment of highly metastatic cell lines

To establish highly metastatic cell lines, cervical lymph node tissues from tumor-bearing mice were crushed using a biomasher (Nippi Biomasher II; Nippi Corporation, Tokyo, Japan) and seeded into cell culture dishes. Metastatic cancer cells that formed colonies were collected, passaged 2–3 times, and inoculated into mouse tongues.

### In vitro proliferation, migration, and invasion assays

Cancer cells (1 × 10^4^ cells/mL) were seeded in 96-well plates. Cells were cultured in a CO_2_ incubator as described above, and cell proliferation was determined every 24 h using Cell Counting Kit-8 (CCK-8; Dojindo Molecular Technologies, Inc., Kumamoto, Japan). To each well, 10 μL CCK-8 reagent was added, and cells were incubated at 37 °C for 1 h. The optical density values were determined using the Bio-Rad iMark™ microplate absorbance reader (Bio-Rad Laboratories Inc., Hercules, CA, USA) at 450 nm. Each assay was performed in triplicates.

For the migration assay, Falcon Cell Culture Inserts (Corning, NY, USA) were utilized, employing a Transwell chamber with 8-μm pores. Cells were suspended in DMEM Ham’s F-12 at a concentration of 4 × 10^5^ cells/mL and then seeded with 500 µL into the upper chamber (2 × 10^5^ cells). The lower chamber was filled with 750 µL DMEM Ham’s F-12 with 10% FBS and incubated for 72 h. To inhibit cell growth, cytosine βD-arabinofuranoside hydrochloride (10 μM; Sigma-Aldrich, St Louis, MO, USA), a selective inhibitor of DNA synthesis that does not inhibit RNA synthesis, were added in both chambers. Cells that migrated to the reverse side were fixed in 4% paraformaldehyde phosphate buffer (4% PFA: FUJIFILM Wako Pure Chemicals Corporation, Osaka, Japan) for 10 min and stained with Mayer’s Hematoxylin (FUJIFILM Wako Pure Chemicals Corporation, Osaka, Japan) for 30 min. The membrane was observed under a microscope (Leica DM IL LED; Leica Microsystems GmbH, Wetzlar, Germany) at 200x, 15 fields of view were taken at random, and the number of cells passing through the membrane was counted.

For the invasion assay, Corning BioCoat Matrigel Invasion Chamber (Corning, NY, U.S.A.) was utilized, employing a Transwell chamber with 8-μm pores coated with Matrigel. Cells were suspended in DMEM Ham’s F-12 at a concentration of 4 × 10^5^ cells/mL and then seeded with 500 µL into the upper chamber (2 × 10^5^ cells). The lower chamber was filled with 750 µL DMEM Ham’s F-12 with 10% FBS and incubated for 72 h. To inhibit cell growth, cytosine βD-arabinofuranoside hydrochloride (10 μM), were added in both chambers. Staining and analysis were performed in the same manner as in the migration assay.

### The preparation of RNA and RT-qPCR method

Total RNA was extracted from the cultured cells and tissues using a total RNA isolation system (Invitrogen PureLink RNA Mini Kit, Invitrogen, CA, USA). ReverTra Ace® qPCR RT Master Mix (Toyobo Co., Ltd., Osaka, Japan) was used for reverse transcription reaction to synthesize single-stranded cDNAs. Quantitative real-time PCR (qPCR) was performed using the SYBR Green PCR protocol with the THUNDERBIRD Next SYBR qPCR Mix (Toyobo Co., Ltd., Osaka, Japan) and a StepOnePlus™ Real-Time PCR system (Applied Biosystems, NJ, USA). The thermocycling conditions for qPCR were as follows: initial denaturation at 95 °C for 20 s, followed by 40 cycles of 95 °C for 3 s and 60 °C for 30 s for annealing and extension. Subsequently, melt curve step was added. The SYBR Green primers used for amplification are listed in Supplementary Table 1.

### Western blotting

Cells and tissues were solubilized in lysis buffer (Ez RIPA Lysis kit; ATTO Corporation, Tokyo, Japan) to obtain whole cell lysates. The lysates were centrifuged at 4 °C at 15,000×*g* for 10 min, and the supernatants were heated with SDS sample buffer (Ez Apply: ATTO Corporation, Tokyo, Japan) at 95 °C for 5 min. Proteins were separated based on their molecular weights using sodium dodecyl sulfate–polyacrylamide gel electrophoresis (SDS-PAGE: ATTO Corporation, Tokyo, Japan) and transferred onto polyvinylidene fluoride membranes. Immunoblotting with primary antibodies and detection using horseradish peroxidase-coupled anti-rabbit IgG antibody and Super Signal™ West Pico PLUS Chemiluminescent Substrate (Thermo Fisher Scientific, MA, USA) Gene Gnome (SynGene, Cambridge, UK) were employed to capture images. We used ImageJ to quantitatively evaluate the results. The antibodies used are listed in Supplementary Table 2.

### Immunocytochemistry

In removable 12-well chambers (ibidi, Gräfelfing, Germany), 5 × 10^4^ cells were seeded into each chamber. After incubation for 24 h, cells were fixed with a 4% PFA solution or 100% methanol (FUJIFILM Wako Pure Chemicals, Osaka, Japan) and washed with PBS (phosphate-buffered saline solution; FUJIFILM Wako Pure Chemicals Corporation, Osaka, Japan). After washing with PBS and blocking with PBS containing 1.5% bovine serum albumin (fraction V, pH 7.0; FUJIFILM Wako Pure Chemicals Corporation, Osaka, Japan) for 30 min, the slides were incubated with primary antibodies at 4 °C for 12 h (Supplementary Table 2). The slides were then incubated with the secondary antibody, TRITC-conjugated AffiniPure Goat Anti-Rabbit IgG (H + L) (Jackson ImmunoResearch Inc., PA, USA), for 1 h at room temperature (RT). The slides were covered with 4ʹ,6-diamidino-2-phenylindole (DAPI) Fluoromount-G mounting medium (SouthernBiotech, Birmingham, AL, USA).

### Immunohistochemistry

Tongue and cervical lymph nodes excised from carcinoma-bearing mice were fixed in a 4% PFA solution for 24 h, and paraffin and frozen sections were prepared. For immunohistochemistry, paraffin sections were deparaffinized, and antigen retrieval was performed by heating in a pressure cooker in ethylenediaminetetraacetic acid (EDTA) solution (pH = 8) for 2 min or autoclaving in citrate buffer (pH = 6) for 20 min. Endogenous peroxidase activity was blocked with 3% hydrogen peroxide for 15 min in methanol. After washing in PBS, the sections were exposed to PBS containing 1% bovine serum albumin and 0.05% sodium azide for 1 h to reduce nonspecific antibody binding. The slides were incubated overnight at 4 °C using rabbit polyclonal antibody (1:500; Invitrogen). After washing with PBS, the slides were incubated at RT with a secondary antibody for 1 h. Primary antibodies were visualized using ImmPACT NovaRED (VECTOR LABORATORIES, CA, USA). Finally, the sections were counterstained with hematoxylin.

Paraffin-embedded or frozen sections were used for fluorescent immunohistochemistry. Paraffin sections were deparaffinized, and antigen retrieval was performed as in immunohistochemistry. Frozen sections were washed with PBS solution and exposed to PBS containing 1% bovine serum albumin and 0.05% sodium azide for 1 h to reduce nonspecific antibody binding. The slides were incubated overnight at 4 °C using rabbit polyclonal antibody (1:500; Invitrogen). After washing with PBS, the slides were incubated at RT with a secondary antibody for 1 h. TRITC-conjugated anti-rabbit IgG antibody, diluted 1:100 (Jackson ImmunoResearch Inc.), was used as the secondary antibody and encapsulated with DAPI Fluoromount-G encapsulant. For double staining, the primary antibodies were labeled using a labeling kit (FlexAble CoraLite® Plus 488 or 555 Antibody Labeling Kit for Rabbit IgG: Proteintech, NJ, USA).

After staining, the sections were observed under a microscope at 400x magnification, and the stained areas were photographed in five views and quantitatively evaluated using ImageJ. Positive density was measured for Cd31, Lyve-1, COX-2, αSMA, and pSTAT3; Cd45-, F4/80-, and Cd163-positive cells were counted. Antibodies used in this study are listed in Supplementary Table 2.

### Inflammation antibody array

Proteins were extracted from tongue tumor tissues of carcinoma-bearing mice and the expression of 40 inflammation-related cytokines and chemokines was comprehensively detected using the Mouse Inflammation Antibody Array G Series 1 (RayBio, GA, USA).

### Enzyme-linked immunosorbent assay (ELISA)

Proteins were extracted from tongue tumor tissues of carcinoma-bearing mice, and the expression levels of RANTES were detected using the Mouse/Rat CCL5/RANTES Quantikine ELISA Kit (R&D Systems, Minneapolis, MN, USA), according to the manufacturer’s instructions.

### Experiments with CCL5

Cancer cells (15 × 10^4^) were seeded onto 6-well plates and cultured for 1 d. Subsequently, 1 μg/mL CCL5 (Recombinant Murine RANTES (CCL5): Proteintech) was added, and the cells were incubated for 24 h in a 5% CO_2_ gas phase. RNA and proteins were extracted.

### Experiments with IL-6 and STAT3 inhibitor

Cancer cells (1 × 10^5^) were seeded onto 6-well plates and cultured for 2 d. Subsequently, 25 ng/mL and 50 ng/mL of interleukin-6 (IL-6, mouse, recombinant, animal-derived free: FUJIFILM Wako Pure Chemicals Corporation, Osaka, Japan) were added. RNA and protein were extracted by incubation for 24 h in a 5% CO_2_ gas.

A STAT3 inhibitor, C188-9 (Med Chem Express, NJ, USA), was prepared at a concentration of 10 µM and added to the cells along with 50 mg/mL IL-6. The cells were then incubated for 24 h in a 5% CO_2_ vapor phase, after which RNA and protein were extracted.

### Statistical analysis

Data of tissue staining are presented as mean ± standard error of mean (SEM); other data are presented as mean ± standard deviation (SD) and generated with a minimum of three biological and technical replicates. Student’s t-test was used to compare two independent groups, Fisher’s exact probability test was used to analyze 2 × 2 contingency tables, and one-way analysis of variance (ANOVA) and Dunnett’s multiple comparison test were used for statistical analyses. Analyses were performed using Prism 10 software (GraphPad Software, La Jolla, CA, USA). Statistical significance was defined as *p* < 0.05.

## Results

### Establishment of the highly metastatic NR-S1-LM4 cell line

We established a highly metastatic cell line to analyze cervical lymph node metastasis in a syngeneic mouse model using normal immune C3Hf/He mice. Briefly, 1 × 10^6^ NR-S1 (Pa) cells were inoculated into the tongue of C3Hf/He mice, and the tongue tumor size increased 30 d later. Extracted cervical lymph nodes were cultured and re-inoculated into the mouse tongue as described in the “Materials and Methods” section. This step was repeated four times to establish NR-S1-LM4 (LM4) cells (Fig. 1b). Although there was no change when comparing cells that had undergone this step three times with Pa cells, LM4 cells had a slightly spindle-shaped morphology compared with Pa cells (Fig. 1c). LM4 cells showed no change in cell proliferative capacity compared to Pa cells (Fig. 1d), whereas migration (Fig. 1e) and invasion (Fig. 1f) capacities significantly increased. After establishing LM4 cells, STR analysis was performed on both the Pa cells and the LM4 cells, and it was confirmed that they were derived from the same cell line (Supplementary Fig. 1).

### Relationship between LM4 cells and EMT

**Fig. 2:**
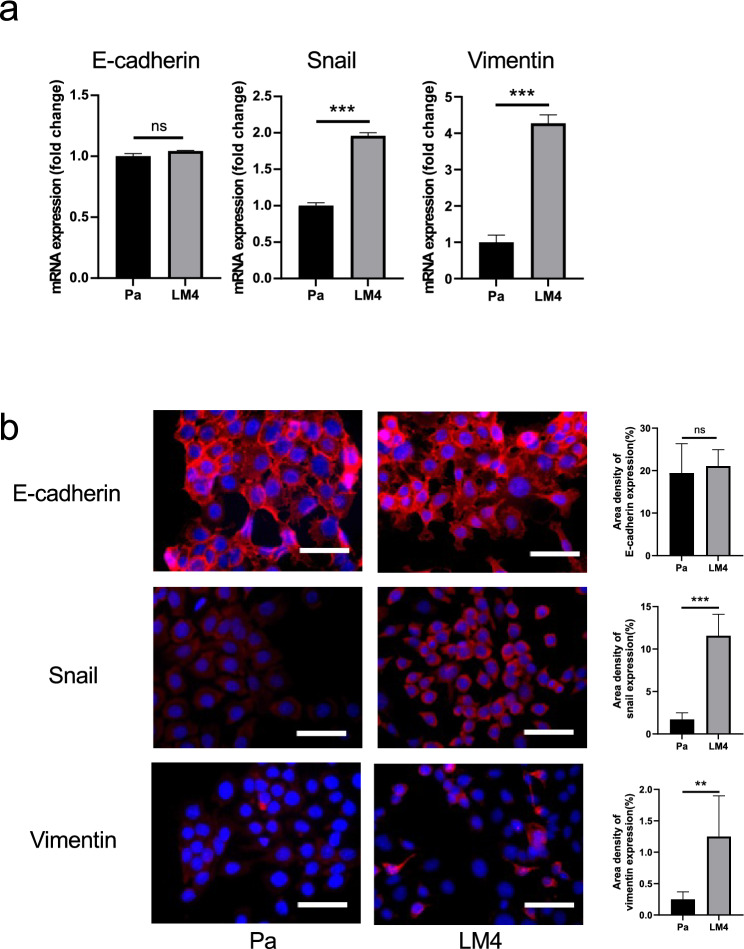
EMT-related marker expression in Pa and LM4 cells. **a** mRNA expression levels of the EMT-related markers E-cadherin, vimentin, and Snail are demonstrated in LM4 cells compared to in Pa cells. The expression levels of each gene were corrected for Gapdh expression and are shown as a multiple of the expression levels in the control group. Values represent mean and standard deviation. n = 3. ***p < 0.001. **b** Immunocytochemical staining of EMT-related markers is shown; protein expression of E-cadherin was unchanged, but protein expression of vimentin and Snail increased in LM4 cells compared to in Pa cells. Red: E-cadherin (top row), Snail (middle row), Vimentin (bottom row) Quantitative data are shown on the right; blue: DAPI. Scale bar: 50 µm. n = 5. **p < 0.01, ***p < 0.001. (Colour figure online)

Because of the morphological differences between Pa and LM4 cells, we considered the possibility that EMT was induced in LM4 cells and compared the expression of EMT-related markers in Pa and LM4 cells at the mRNA level. Additionally, we compared the expression of EMT-related markers at the protein level using immunocytochemistry. The results showed that the epithelial marker E-cadherin was not significantly altered at the mRNA or protein levels, whereas the expression levels of the mesenchymal markers vimentin and Snail were significantly higher in LM4 cells than in Pa cells (*p* < 0.05; Fig. 2). Recently, the concept of hybrid EMT has been proposed in which both epithelial and mesenchymal markers are expressed, suggesting that highly metastatic lymph node cell lines are in a hybrid EMT state [33, 34].

### Investigation of tumor tissue and lymph node metastatic potential using a syngeneic mouse model of oral cancer

**Fig. 3:**
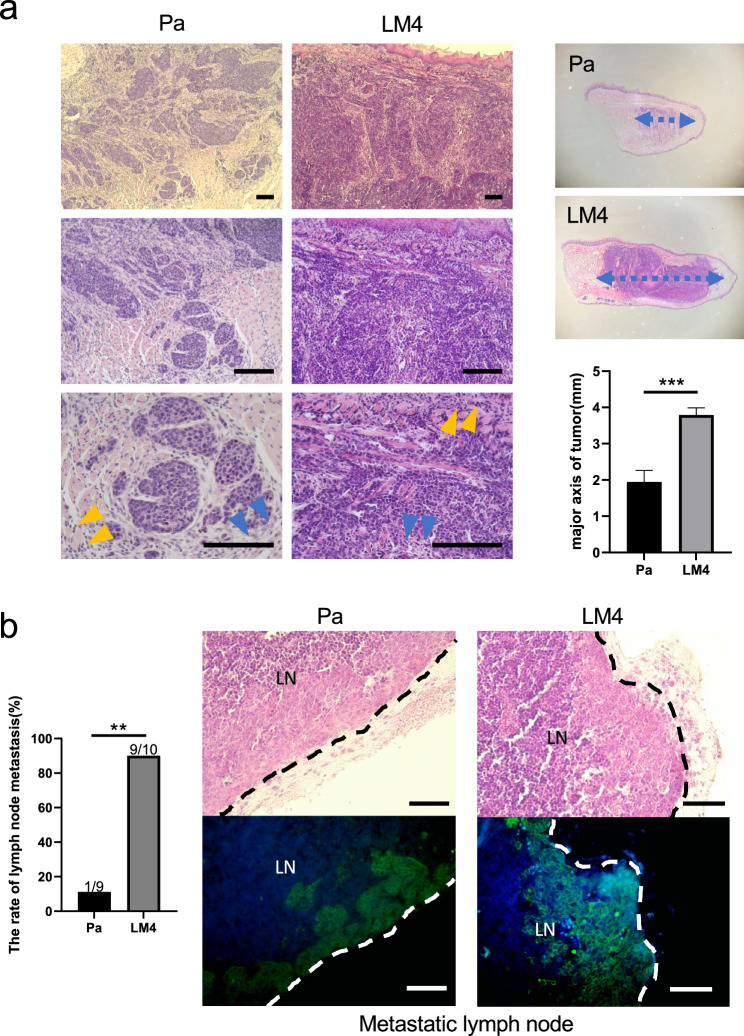
Comparison of histology of Pa and LM4 tumors. **a** Hematoxylin and eosin (H&E) stained images of Pa and LM4 tumors are shown; Pa tumors showed multiple focal tumor masses with fibroblast and collagen fiber growth around them and lymphocyte infiltration between tumor masses. LM4 tumors, on the other hand, are fully grown with histology in which collagen fibers were embedded between the cancer cells. An infiltration of lymphocytes was observed around and inside the tumor. A graph comparing tumor length diameter is shown on the right: compared to Pa tumors, LM4 tumors had a significantly larger tumor length diameter; Pa tumor group: n = 7, LM4 tumor group: n = 10; scale bar: 50 µm; ***p < 0.001. Blue arrows: fibroblast and collagen fiber, yellow arrows: lymphocyte. **b** Cervical lymph node metastasis was observed in one of nine individuals in the Pa tumor group and nine of ten individuals in the LM4 tumor group; H&E staining and fluorescent immunohistochemistry of Pan-cytokeratin are shown. Pan-cytokeratin-positive cancer cells were found in the lymph nodes, confirming cervical lymph node metastasis; Pa tumor group: n = 9; LM4 tumor group: n = 10; blue: DAPI; green: Pan-cytokeratin; scale bar: 50µm; **p < 0.01

We examined the metastatic potential of Pa and LM4 cells in a syngeneic mouse model of oral cancer. C3Hf/He mice were inoculated with 5 × 10^5^ Pa or LM4 cells in the tongue. After 14 days, the tongue tumors and cervical lymph nodes were removed. The Pa tongue tumors showed multiple small foci-like tumor masses with proliferating fibroblasts and collagen fibers, and lymphocytes were observed around the tumor masses (Fig. 3a). In contrast, the LM4 tongue tumor showed full growth, with a histological appearance in which collagen fibers were embedded between cancer cells, and an infiltration of lymphocytes was observed around and inside the tumor (Fig. 3a). These results suggest that although both Pa and LM4 tumors have inflammation in the tumor tissue, LM4 tumors may have increased inflammation in their interior. In addition, the length and diameter of LM4 tumors was considerably larger than that of the Pa tumors (Fig. 3a). This showed that the in vivo tumor growth rate of LM4 was faster than that of Pa and that LM4 cells had a higher tumor-forming capacity.

Next, the presence of cervical lymph node metastasis was analyzed using hematoxylin and eosin staining and fluorescent immunohistochemistry for Pan-cytokeratin in the excised cervical lymph nodes. Fourteen days after inoculation, cervical lymph node metastasis was observed in one out of nine animals in the Pa-inoculated group and nine out of ten animals in the LM4 inoculated group, showing a markedly higher rate of cervical lymph node metastasis in the LM4 inoculated group (Fig. 3b). Several lymph nodes in LM4 tumor-bearing mice showed histological findings suggestive of extracapsular invasion (Supplementary Fig. 2).

### Immunohistological investigation of tongue tumors using a syngeneic mouse model of oral cancer

**Fig. 4:**
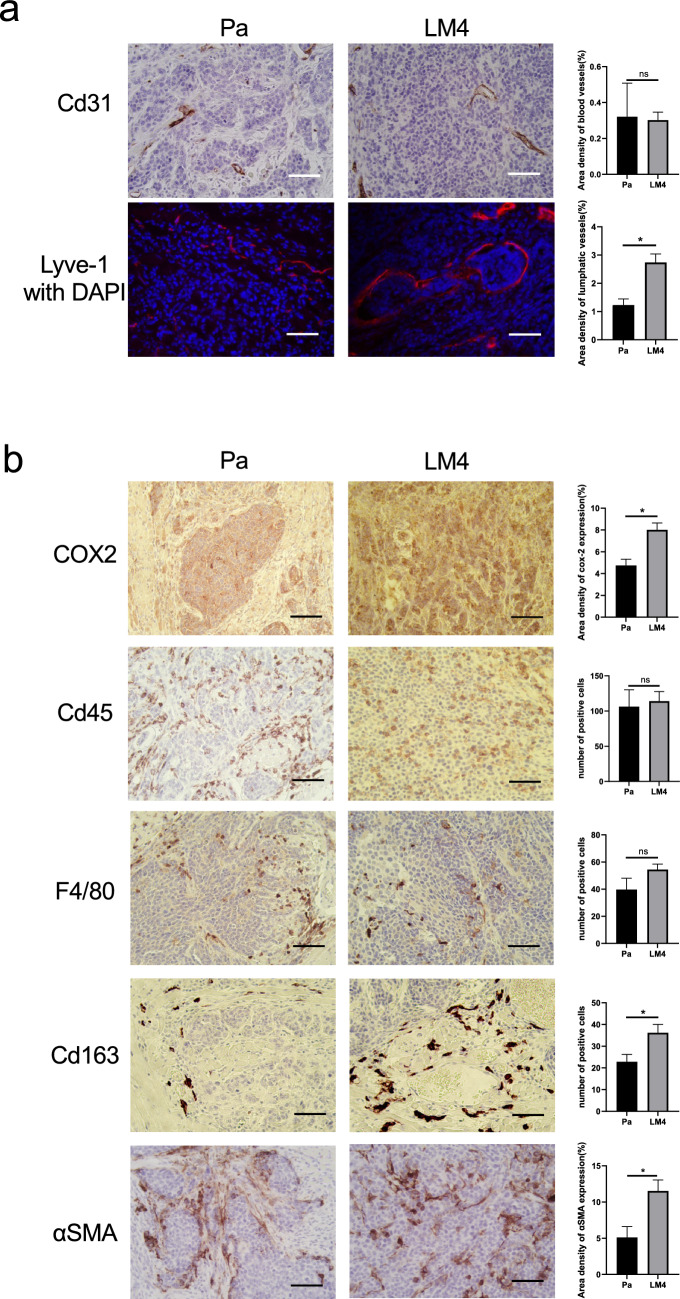
Immunohistological comparison of Pa and LM4 tumors. **a** Immunohistochemistry and fluorescence immunohistochemistry images of Pa and LM4 tumor tissues are shown; Cd31-positive vascular endothelial cells were not significantly different between Pa and LM4 tumors; Lyve-1-positive lymphatic endothelial cells were significantly increased in LM4 tumors compared with in Pa tumors. Quantitative data are shown on the right; five fields of view were taken, and the mean positive area per field of view was calculated. Pa tumors: n = 7; LM4 tumors: n = 10; scale bar: 50 µm; *p < 0.05. **b** Immunohistological staining images of Pa and LM4 tumor tissues showing significantly increased COX-2 expression in LM4 tumors compared to in Pa tumors. In addition, the number of Cd45-positive leukemic cells and F4/80-positive macrophages were not significantly different, while the number of Cd163-positive M2-TAMs significantly increased in LM4 tumors compared to in Pa tumors. In addition, αSMA-positive CAFs significantly increased in LM4 tumors. Quantitative data are shown on the right. For COX-2 and αSMA, five views were taken, and the mean positive area per view was calculated; for Cd45, F4/80, and Cd163, five views were taken, and the mean positive cell count per view was calculated. Pa tumors: n = 7; LM4 tumors: n = 10; Scale bar: 50 µm. *p < 0.05

Pa and LM4 tumors were examined immunohistologically. Angiogenesis in tumor tissues is crucial for tumor growth [35]. However, immunohistochemical staining for Cd31, a marker of vascular endothelial cells, showed no significant change in the area of blood vessels per tumor area in LM4 tumors compared to Pa tumors (Fig. 4a). By contrast, lymphangiogenesis is important for establishing lymph node metastasis [29].

Fluorescent immunohistochemistry for Lyve-1, a marker of lymphatic endothelial cells, revealed a remarkable increase in lymphatic vessels in LM4 tumors compared to Pa tumors (Fig. 4a).

Subsequently, we investigated Cyclooxygenase-2 (COX-2) expression, which is induced at inflammatory sites and is associated with lymph node metastasis, oral cancer prognosis, and EMT induction. Moreover, Cd45, as a marker of leukocytes; F4/80, as a marker of tumor-associated macrophages (TAM); and Cd163, as a marker of M2-TAMs, have been investigated immunohistologically [36–38]. The results showed that COX-2 expression was higher in LM4 tumors (Fig. 4b). Furthermore, we found no significant differences in the number of leukocytes or TAMs; however, M2-TAMs were considerably increased in LM4 tumors, mainly in the peritumoral stroma (Fig. 4b). In addition, αSMA, as a marker for cancer-associated fibroblasts (CAFs), levels were evaluated. We observed that aSMA levels notably increased in LM4 tumors, especially in the histology αSMA-positive cells invading the tumor interior (Fig. 4b).

### Comparison of inflammatory cytokine expression in the TME

**Fig. 5:**
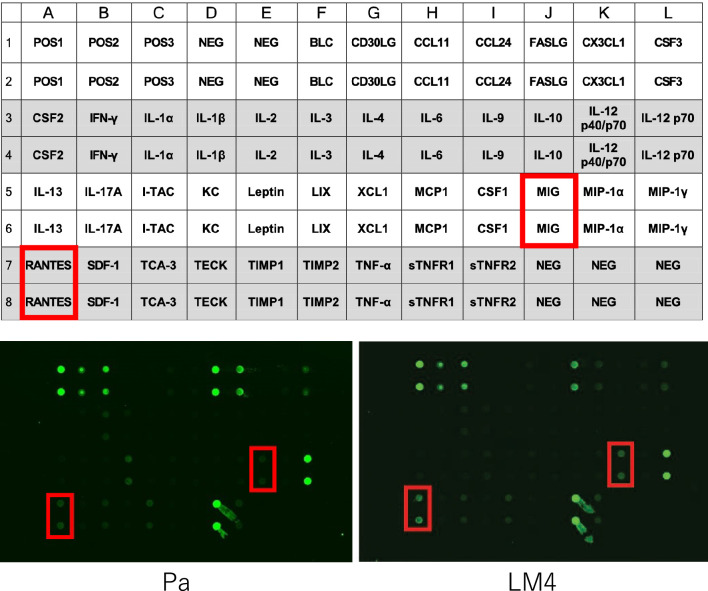
Inflammation antibody array. Forty inflammation-related cytokines and chemokines are listed. CCL5 and CXCL9 were upregulated in LM4 tumors compared to in Pa tumors

**Fig. 6:**
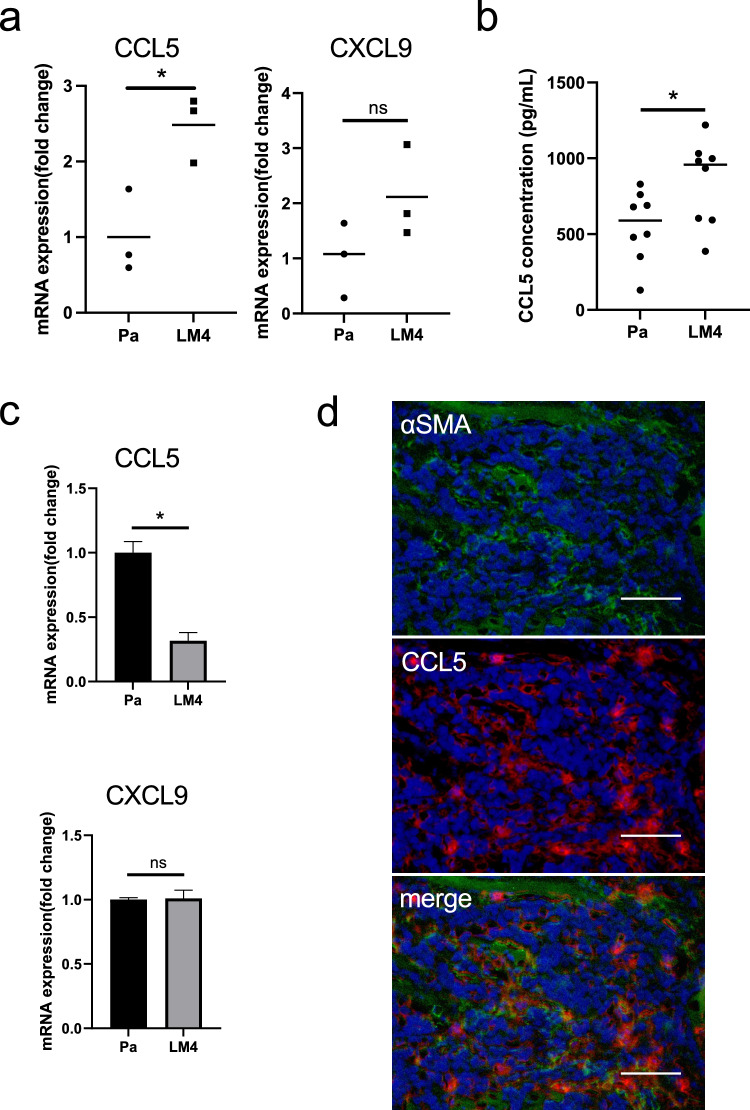
Examination of inflammatory cytokine expression. **a** RNA extracted from Pa and LM4 tumor tissues was used to compare mRNA expression levels within each tumor by RT-qPCR. mRNA expression levels are shown. The expression levels of each gene were corrected for Gapdh expression and expressed as a multiple of the expression level in the Pa tumor group. The mean of each group is represented by a line. n = 3. *p < 0.05. **b** Protein extracted from Pa and LM4 tumor tissues was used to compare CCL5 expression levels within each tumor by ELISA. Protein expression levels are shown. The expression levels of each gene were corrected for Gapdh expression and expressed as a multiple of the expression level in the Pa tumor group. The mean of each group is represented by a line. n = 8. *p < 0.05. **c** RNA extracted from Pa and LM4 culture cells was used to compare mRNA expression levels by RT-qPCR. mRNA expression levels are shown. The expression levels of each gene were corrected for Gapdh expression and expressed as a multiple of the expression level in the Pa cells group. Values represent mean and standard deviation. n = 3. *p < 0.05. **d** Tissue immunofluorescence assay suggested that CCL5 (Red) and the CAF marker αSMA (Green) were co-localized in LM4 tumors. Scale bar, 10 μm. (Colour figure online)

As increased expression of COX-2 and TAM was observed, the expression of inflammatory cytokines in whole tumor tissues was comprehensively investigated. Proteins were extracted from Pa and LM4 tumor tissues and an inflammation antibody array was performed. Of the 40 cytokines screened, CCL5 (RANTES) and CXCL9 (MIG) were upregulated in LM4 tumors compared to in Pa tumors (Fig. 5, Table 1). To confirm the anti-inflammatory antibody array results at the mRNA level, RNA was extracted from Pa and LM4 tongue tumor tissues, and the expression of CCL5 and CXCL9 was analyzed using RT-qPCR. The results were similar to those for CCL5, but not CXCL9, in the inflammation antibody array (Fig. 6a). Furthermore, CCL5 protein expression levels were confirmed in Pa and LM4 tongue tumor tissues using ELISA. CCL5 expression was higher in LM4 tumors than in Pa tumors (Fig. 6b). In contrast, CCL5 and CXCL9 expression in Pa and LM4 cancer cells was compared at the mRNA level to confirm whether CCL5 and CXCL9 are secreted by cancer cells in vitro (Fig. 6c). The results showed that CCL5 expression was reduced in LM4 cells, whereas CXCL9 expression did not differ between Pa and LM4 cells. These data suggest that CCL5 is secreted in large quantities from cells other than LM4 tumors.

**Table 1:** The list of the results of the inflammation antibody array

Description	Normalization data		
	NR-S1-LM4	NR-S1-Pa	Ratio
sTNF RI	25,451.91	20,982.00	1.2130
MIP-1g	22,770.50	23,969.50	0.9500
Eotaxin	4575.29	13,412.00	0.3411
RANTES	2808.55	1293.50	2.1713
MIG	2286.66	702.50	3.2550
GCSF	1209.37	1187.00	1.0188
sTNF RII	1080.25	797.00	1.3554
TIMP-1	1051.67	1076.00	0.9774
TCA-3	826.45	663.00	1.2465
Eotaxin-2	817.58	5094.00	0.1605

The results of analysis using RayBio® Mouse Inflammation Antibody Array 1 (G-Series) are shown. Listed are 10 proteins that were highly expressed in NR-S1-LM4 cells

Considering the possibility that CCL5 is secreted from TME component cells rather than LM4 cells and the increasing CAFs in LM4 tumors compared with Pa tumors, immunohistochemistry was performed on αSMA, a marker for CAFs, and CCL5 (RANTES). The same sites were stained, suggesting that CCL5 was secreted from CAFs (Fig. 6d). These data suggest that CCL5 is secreted by CAFs and may contribute to the increased aggressiveness of LM4 tumors.

### Effect of CCL5 on the STAT3 signaling pathway

**Fig. 7:**
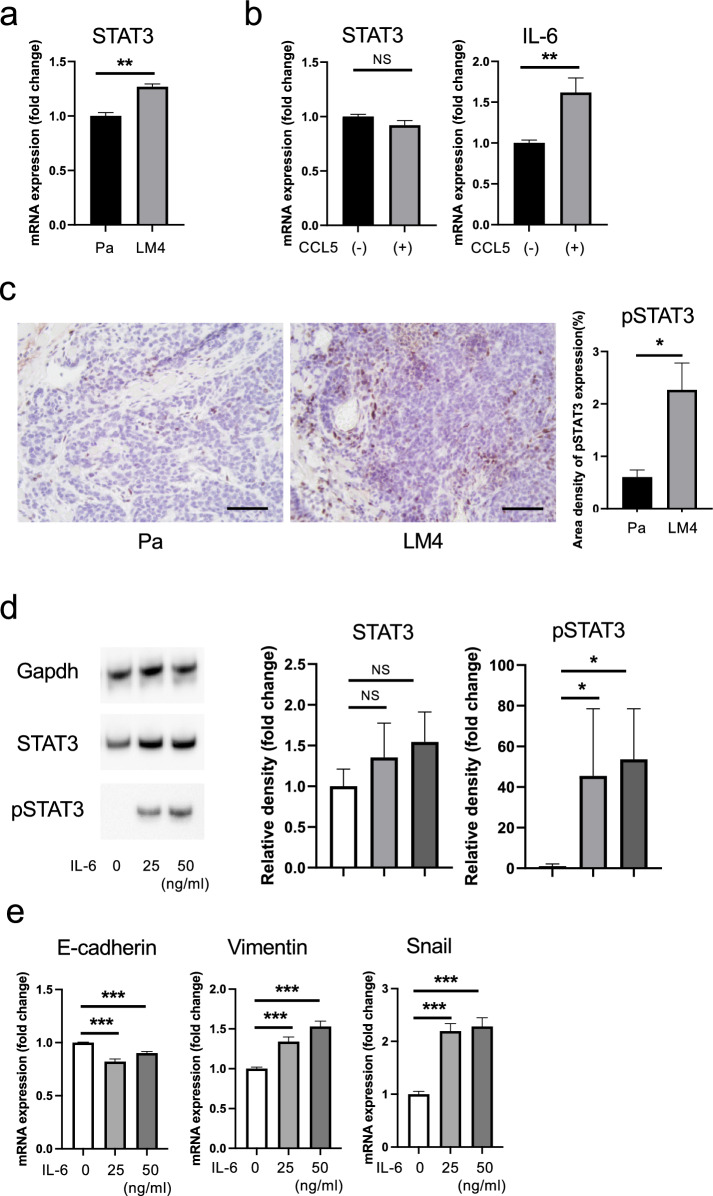
Effects of CCL5 and IL-6 on STAT3 activation and EMT-related markers in LM4 cells. **a** The STAT3 mRNA expression of Pa and LM4 cells was compared using RT-qPCR. mRNA expression, corrected using Gapdh expression, is expressed as a multiple of the expression in the Pa group. Values represent mean and standard deviation. n = 3. **p < 0.01. **b** CCL5 was added to LM4 cells. After incubation for 24 h, STAT3 and IL6 mRNA expression was compared using RT-qPCR. mRNA expression, corrected using Gapdh expression, is expressed as a multiple of the expression in the Pa group. Values represent mean and standard deviation. n = 3. **p < 0.01. **c** Immunohistological staining images of Pa and LM4 tumor tissues showing significantly increased pSTAT3 expression in LM4 tumors compared to in Pa tumors. Quantitative data are shown on the right. Five views were taken, and the mean positive area per view was calculated. Pa tumors: n = 3; LM4 tumors: n = 3; scale bar: 50 µm. *p < 0.05. **d** IL-6 was added to LM4 cells. After incubation for 24 h, STAT3 and pSTAT3 protein expression was compared using western blotting. Anti-Gapdh antibody (top panel) was used as an internal control. Quantitative data are shown on the right. n = 4. *p < 0.05. **e** IL-6 was added to LM4 cells. After incubation for 24 h, the mRNA expression of the EMT-related markers E-cadherin, Vimentin, and Snail was measured. mRNA expression, corrected using Gapdh expression, is shown as a multiple of the expression levels in the control group. Values represent mean and standard deviation. n = 3. ***p < 0.001

CCL5 is associated with the STAT3 signaling pathway in several types of cancer [39, 40]. Interestingly, STAT3 mRNA expression was higher in LM4 cancer cells than in Pa cancer cells (Fig. 7a). IL6 is required for STAT3 phosphorylation [41–43]. To investigate how CCL5 secreted from non-tumor cells affects the STAT3 signaling pathway, recombinant CCL5 was added to cancer cells, and changes in STAT3 and IL6 mRNA and protein expression were observed. For LM4 cells, RT-qPCR analysis revealed that STAT3 mRNA expression was almost unchanged, while IL6 expression was elevated, following CCL5 addition (Fig. 7b). In immunohistological analysis, pSTAT3 expression was significantly higher in LM4 tumors than in Pa tumors (Fig. 7c). To examine the effects of STAT3 phosphorylation, we experimentally added IL-6 to LM4 cells. Following IL-6 addition, these cells exhibited STAT3 phosphorylation (Fig. 7d) and, interestingly, enhanced EMT (Fig. 7e).

### Effect of STAT3 phosphorylation inhibition on EMT

**Fig. 8:**
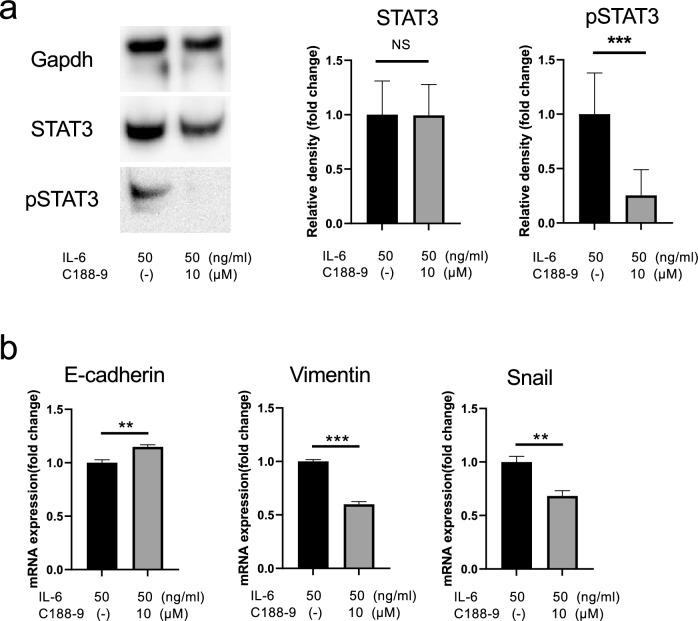
Modulation of IL-6-induced STAT3 activation and EMT-related gene expression by inhibiting STAT3 phosphorylation using C188-9 in LM4 cells. **a** IL-6 was added to LM4 cells in the presence and absence of C188-9, and proteins were extracted after incubation for 24 h. Protein expression of STAT3 and pSTAT3 was compared using western blotting. Anti-Gapdh antibody (top panel) was used as an internal control. Quantitative data are shown on the right. n = 4. ***p < 0.001. **b** IL-6 was added to LM4 cells in the presence and absence of C188-9. After incubation for 24 h, mRNA expression levels of the EMT-related markers E-cadherin, Vimentin, and Snail were measured and demonstrated. The expression levels of each gene were corrected for Gapdh expression and are shown as a multiple of the expression levels in the control group. Values represent mean and standard deviation. n = 3. **p < 0.01, ***p < 0.001

Finally, we investigated the effect of STAT3 phosphorylation inhibition on EMT using the STAT3 phosphorylation inhibitor C188-9 in LM4 cells. Western blotting confirmed that the addition of C188-9 to LM4 cells treated with IL-6 inhibited STAT3 phosphorylation in LM4 cells (Fig. 8a). Furthermore, C188-9 treatment increased E-cadherin expression and considerably decreased Vimentin and Snail expression in LM4 cells (Fig. 8b). These results suggest that STAT3 phosphorylation promotes the induction of EMT in LM4 cells.

## Discussion

The association between inflammation and cancer was first suggested by Rudolf Virchow in the mid-nineteenth century when he reported that cancer develops at sites of chronic inflammation and that tumor biopsy specimens contain large numbers of inflammatory cells [44]. Inflammation is an important hallmark of cancer and is reportedly associated with the risk of tumor development and progression in many types of cancer [17, 18, 45]. Unlike wound healing and infection, the inflammatory response in cancer is difficult to control [46]. Exogenous inflammation in cancer is reportedly caused by autoimmune diseases, bacterial and viral infections, obesity, smoking, asbestos exposure, and excessive alcohol consumption, which promote cancer development and progression. Tumor-induced endogenous inflammation is also known to be caused by mutations involved in cancer development and contributes to tumor progression through the mobilization and activation of inflammatory cells [47–49]. Both exogenous and endogenous inflammation result in an immunosuppressive TME and provide favorable conditions for tumorigenesis [48]. Once an inflammatory TME is formed, inflammatory factors derived from tumor and stromal cells induce tumor cell proliferation and survival by activating oncogenes and tumor suppressor genes [21, 48, 49]. In this study, both Pa and LM4 tumors with different metastatic and tumor growth potentials showed an influx of CD45-positive cells and COX-2 expression in the tumor tissue, resulting in tumor tissue inflammation. In particular, COX-2 expression was elevated, and CAFs were increased in LM4 tumors, confirming that inflammation was enhanced in these tumors. It has been previously reported that COX-2 expression is associated with lymphangiogenesis and cervical lymph node metastasis in studies using immunodeficient mice and human clinical specimens [36, 50]. In the syngeneic mouse model used in this study, lymph node metastasis was observed more frequently in LM4 tumors than in Pa tumors, and COX-2 expression and lymphatic vessels in primary tumor tissues increased, suggesting that this model is a highly useful model that could replicate previous reports. Inflammation induces EMT in cancer cells. In the present study, we found increased inflammation, lymphangiogenesis, M2-TAM, and CAFs in tumor tissues composed of cancer cells that originally promoted EMT induction, and CCL5 and IL-6 secreted by the inflammation further enhanced EMT induction in cancer cells. This suggests the possibility of a synergistic effect between EMT induction and inflammation, although the detailed mechanism is unknown [51–53].

EMT plays an important role in cancer progression and metastasis [54]. However, a simple classification of cancer cells into epithelial and mesenchymal phenotypes has shown contradictory results in various reports, and the concept of a state between epithelial and mesenchymal phenotypes, or hybrid-type EMT, has recently been proposed [33, 34]. Hybrid EMT is considered an alteration in which individual cells express both epithelial and mesenchymal markers to allow cancer cells to flexibly adapt to the stressful environment during the process of metastasis establishment, and the hybrid EMT state is considered more important for cancer metastasis than the complete epithelial or mesenchymal state [34, 55]. In this study, LM4 cells, a highly metastatic lymph node cell line established by in vivo selection using a syngeneic mouse model, did not show decreased expression of the epithelial marker E-cadherin but considerably increased expression of the mesenchymal marker vimentin and Snail. This suggested that the cells were in a hybrid EMT state.

Inflammation mobilizes fibroblasts, induces fibrosis, senses changes in tissue structure caused by tumorigenesis, and produces inflammatory mediators [56]. CAFs, fibroblasts present in the TME, are involved in the deposition of collagen and various ECM components in the TME and are known to promote cancer cell growth and vascular angiogenesis [57, 58]. CAFs also produce numerous cytokines and chemokines such as osteopontin, CXCL1, CXCL2, CXCL12, CXCL13, IL-6, IL-1β, and CCL5, which are important for immune functions [21, 59, 60]. Furthermore, TGF-β secreted by CAFs inhibits the activation of natural killer (NK) cells and CTLs and the differentiation of regulatory T cells (Treg) and immunosuppressive plasma cells [61–63]. In this study, αSMA-positive CAFs were increased in LM4 tumor tissue compared to in Pa tumor tissue, and the presence of CAFs may have contributed to the increased tumor growth rate of LM4 tumors. TAMs, mainly M2-TAMs, cause tumor-promoting inflammation by inhibiting the cytotoxic function of T cells and maintaining the immunosuppressive state of the TME through cytokine secretion [64, 65]. Furthermore, M2-TAMs have been reported to regulate abnormal immune responses, angiogenesis, cell proliferation, and stromal cell remodeling in the TME [66]. It is also known that changes in TAM polarity are caused by stimuli and signals from cancer cells, T cells, and B cells [21, 66]. In the present study, a comprehensive analysis of inflammatory cytokines in LM4 and Pa tumors confirmed that CCL5 expression was elevated in LM4 tumors. Therefore, we focused our analysis on CCL5 expression in several mice and found that CCL5 was considerably upregulated in LM4 tumors at both protein and mRNA levels. CCL5 has been reported to induce M2-TAMs, which is consistent with the increased expression of M2-TAMs in LM4 tumors [67]. In vitro, CCL5 mRNA expression in LM4 cells themselves was rather decreased. This suggests that CCL5, which was upregulated in LM4 tumor tissues, is secreted from TME component cells such as CAFs, which were also upregulated in LM4 tumor tissues upon immunohistochemical analysis. There are also reports that CCL5 is secreted by CAFs, which is consistent with our results [68]. These results suggest that the interaction between cancer cells and the TME is important in cancer progression as well as in the nature of cancer cells.

IL-6 is a multifaceted cytokine involved in inflammation, is a major inducer of C-reactive proteins, and is predominantly produced by T cells and macrophages [69]. IL-6 is one of the major chemokines present in the serum of patients with head and neck cancer, and elevated IL-6 levels have been reported to independently predict tumor recurrence, decreased survival, and tumor metastasis. Yadav et al. showed that IL-6 induces EMT changes in head and neck tumor cells via activation of the STAT3/Snail signaling pathway in a SCID mouse xenograft model and that STAT3 knockdown markedly reversed the EMT changes [70]. These results indicate that IL-6, STAT3, and Snail are directly associated with the recurrence, metastasis, and decreased survival of patients with head and neck cancer [70–75]. Furthermore, Huang et al. reported that tumor-associated macrophage-derived CCL5 promotes prostate cancer stem cells and metastasis through activation of β-catenin/STAT3 signaling [40]. Moreover, CCL5 has been reported to be involved in cancer metastasis and progression through the STAT3 signaling pathway in breast, bladder, and colorectal cancers [76]. Additionally, STAT3 is a downstream target of IL-6, and in response to IL-6 binding, STAT3 is phosphorylated via JAK2 [77, 78]. Our findings reveal that STAT3 expression was higher in LM4 cells, a highly metastatic cell line, than in Pa cells, and that IL-6 expression was enhanced by CCL5 treatment. Moreover, in LM4 cells, IL-6 stimulation enhanced pSTAT3 expression and promoted EMT, via the IL-6/STAT3 transduction pathway.

As inflammation progresses in vivo, not only in cancer, various types of immune-specific cells (leukocytes and other inflammatory cells) are activated and mobilized to inflammatory sites by signaling pathways that include growth factors, cytokines, and chemokines [79]. In cancer, exogenous and endogenous inflammation lead to the activation of transcriptional regulators such as NF-κβ and STAT3 in tumor cells [17]. These transcriptional regulators subsequently induce the production of soluble inflammatory mediators such as cytokines, chemokines, and COX-2. The infiltrating inflammatory cells mobilized by these inflammatory mediators, especially monocytes, granulocytes, and lymphocytes, further induce the production of proinflammatory cytokines, thereby creating a positive feedback loop that amplifies inflammatory symptoms and perpetuates tumorigenesis [48].

Thus, in cancer, various cytokines, including IL-6 and CCL5, are secreted into the TME at various stages of inflammation, and their complex interactions and secretions may be altered. In this study, we demonstrated the interaction between two important proinflammatory cytokines (CCL5 and IL-6) and cancer cells; however, no change in IL-6 expression was observed in RNA extracted from whole tumors. This suggests that, even if IL-6 expression is the same in the whole tumor tissue, IL-6 may affect cancer cells differently, altering their proliferative, migratory, and invasive capacities due to differences in the distribution of IL6; however, further research is required to clarify the details. In addition, RT-qPCR analysis of RNA extracted from tumor tissues showed that cytokine expression was not constant among individuals, indicating the difficulty of TME analysis involving inflammation in vivo. Further improvements, such as increasing the number of samples, are required.

The results obtained in the experimental model used in this study, such as EMT enhancement in highly metastatic cell lines, and the expression of CCL5, which suggests an association with M2-TAMs in the tumor tissue, are generally consistent with previous reports on inflammation and tumor immunity, indicating that this model is very useful for cancer research, particularly within the TME. Continued analysis, incorporating both the cancer cells themselves and the TME research approach, is crucial for further insights.

## Conclusions

In this study, we used a syngeneic mouse model to establish a highly metastatic lymph node cell line for oral cancer. This cell line showed no change in cell proliferative capacity, but tumor-bearing mice showed not only enhanced lymph node metastatic capacity but also markedly enhanced tumor growth capacity. Further analysis showed that inflammatory cytokines (CCL5 and IL-6) derived from the TME are involved in EMT in cancer cells via the STAT3 signaling pathway. This strongly suggests the importance of the interaction between cancer cells and the TME.

This model, which allows for the use of two types of cells with different metastatic and tumor growth potentials, is very useful for oral cancer research involving the interaction between cancer cells and the TME in tumor tissues and serves as a valuable tool for exploring new therapeutic agents.

Extracapsular invasion of lymph node metastasis was observed in three of ten specimens from the LM4 tumor group. The broken line indicates the capsule of the lymph node, and the arrow indicates extracapsular infiltrated cancer cells. Scale bar: 50 μm.

## Supplementary Information

**Supplementary Table 1.** List of SYBR Green primers used for amplification.

**Supplementary Table 2.** List of antibodies used for immunocytochemistry, western blotting, immunohistochemistry, and immunofluorescence.

**Supplementary Figure 1.** The results of STR analysis for NR-S1 cells (Pa cells) and LM4 cells.

STR profiles of NR-S1 were not completely matched with LM4. But Evaluation value (EV) between NR-S1 and LM4 was 0.94, which was high enough that STR profiles of NR-S1 are the same as those of LM4. Therefore two cell lines were considered to be the identical cell strain.

**Supplementary Figure 2.** Extracapsular invasion of lymph node metastasis.

Below is the link to the electronic supplementary material.

Supplementary file1 (PDF 373 KB)

Supplementary file2 (PDF 37 KB)

## Data Availability

No datasets were generated or analysed during the current study.
