# Supplementary material for: Tumor microenvironment dynamics in oral cancer: unveiling the role of inflammatory cytokines in a syngeneic mouse model
Source: Clin Exp Metastasis. 2024 Aug 10;41(6):891–908. doi: 10.1007/s10585-024-10306-1 (PMC11607012; doi:10.1007/s10585-024-10306-1)
Supplement: Supplementary file 1 — Supplementary file1 (PDF 373 KB) [file 10585_2024_10306_MOESM1_ESM.pdf]

Sample: NR-S1

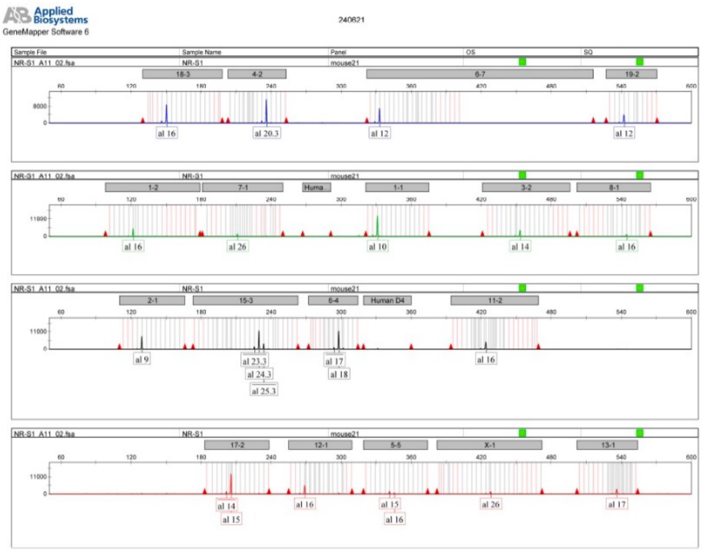

Sample: LM4

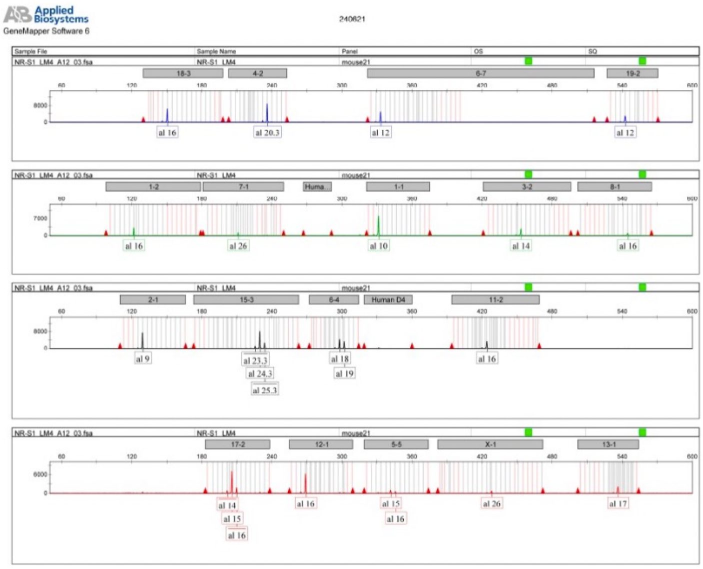

STR Profile

Locus	NR-S1			LM4		
Mouse STR 18-3	16			16		
Mouse STR 4-2	20.3			20.3		
Mouse STR 6-7	12			12		
Mouse STR 19-2	12			12		
Mouse STR 1-2	16			16		
Mouse STR 7-1	26			26		
Mouse STR 1-1	10			10		
Mouse STR 3-2	14			14		
Mouse STR 8-1	16			16		
Mouse STR 2-1	9			9		
Mouse STR 15-3	23.3	24.3	25.3	23.3	24.3	25.3
Mouse STR 6-4	17	18		18	19	
Mouse STR 11-2	16			16		
Mouse STR 17-2	14	15		14	15	16
Mouse STR 12-1	16			16		
Mouse STR 5-5	15	16		15	16	
Mouse STR X-1	26			26		
Mouse STR 13-1	17			17		

Supplementally figure1

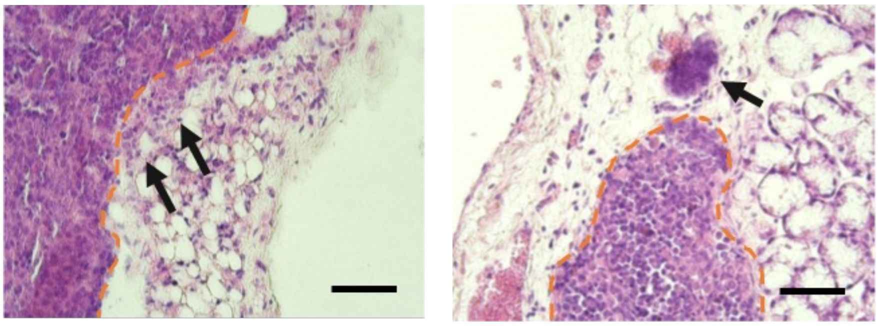

Supplementally figure2
