# Supplementary material for: Tumor microenvironment dynamics in oral cancer: unveiling the role of inflammatory cytokines in a syngeneic mouse model
Source: Clin Exp Metastasis. 2024 Aug 10;41(6):891–908. doi: 10.1007/s10585-024-10306-1 (PMC11607012; doi:10.1007/s10585-024-10306-1)
Supplement: Supplementary file 2 — Supplementary file2 (PDF 37 KB) [file 10585_2024_10306_MOESM2_ESM.pdf]

Gene name		Sequence(5'→3')
E-cadherin	Forward	CAGGTCTCCTCATGGCTTTGC
	Reverse	CTTCCGAAAAGAAGGCTGTCC
Vimentin	Forward	GCTGCGAGAGAAATTGCAGGA
	Reverse	CCACTTTCCGTTCAAGGTCAAG
Snail	Forward	GCTGCGAGAGAAATTGCAGGA
	Reverse	CCACTTTCCGTTCAAGGTCAAG
STAT3	Forward	CAATACCATTGACCTGCCGAT
	Reverse	GAGCGACTCAAACTGCCCT
CXCL9	Forward	TCCTTTTGGGCATCATCTTCC
	Reverse	TTTGTAGTGGATCGTGCCTCG
CCL5	Forward	CCTGCTGCTTTGCCTACATTGC
	Reverse	ACACACTTGGCGGTTCTTTCGG
IL-6	Forward	CTGCAAGAGACTTCCATCCAG
	Reverse	AGTGGTATAGACAGGTCTGTTGG
Gapdh	Forward	AGGTCGGTGTGAACGGATTTG
	Reverse	TGTAGACCATGTAGTTGAGGTCA

Supplementally Table 1

Antibody	Dilution	Company
E-cadherin	ICC(1:200)	Proteintech
Vimentin	ICC(1:100)	Proteintech
Snail1	ICC(1:100)	GeneTex
Cd31	IHC(1:1000)	Proteintech
Lyve1	IF(1:250)	Abcam
COX-2	IHC(1:200)	Proteintech
Cd45	IHC(1:200)	Cell Signaling
F4/80	IHC(1:250)	Cell Signaling
Cd163	IHC(1:500)	Abcam
αSMA	IHC(1:500),IF(1:200)	Cell Signaling
Pan-cytokeratin<br>Alexa Fluor488® conjugated	IF(1:100)	Invitrogen
CCL5	IF(1:50)	Affinity Biosciences
β-tubulin	WB(1:5000)	Proteintech
STAT3	WB(1:2000)	Cell Signaling
pSTAT3	WB(1:2000), IHC(1:100)	Cell Signaling

Supplementally Table 2
